# Supplementary material for: Global, regional, and national burden of osteoarthritis from 1990 to 2021 and projections to 2035: A cross-sectional study for the Global Burden of Disease Study 2021
Source: PLoS One. 2025 May 27;20(5):e0324296. doi: 10.1371/journal.pone.0324296 (PMC12111611; doi:10.1371/journal.pone.0324296)
Supplement: S1 Fig — Abbreviations: OA = osteoarthritis. (DOCX) [file pone.0324296.s001.docx]

**S1 Fig. Trends in the all-age cases and age-standardized incidence and prevalence rates of OA by sex from 1990 to 2021.**


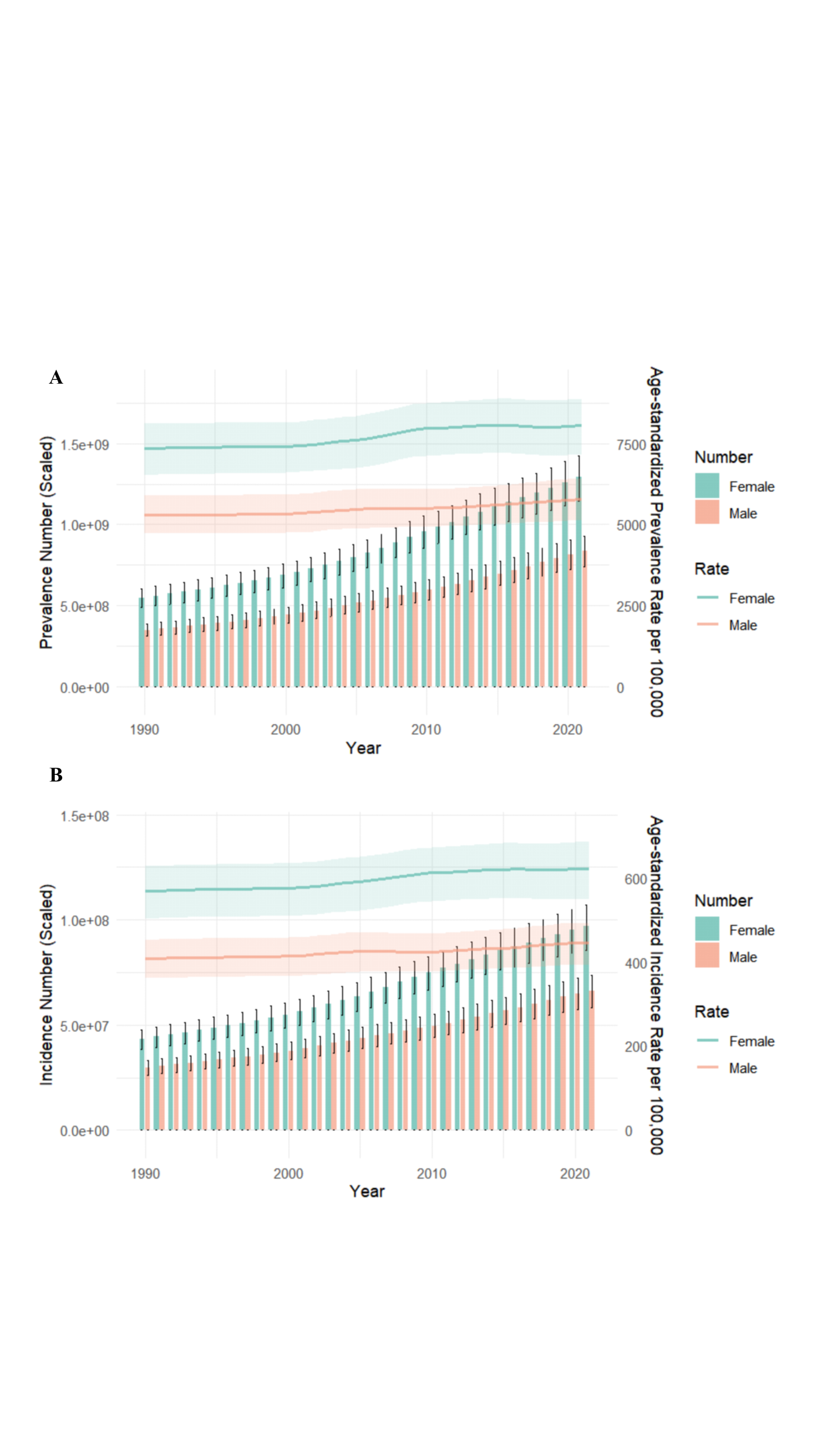


S1 Fig. Trends in the all-age cases and age-standardized incidence and prevalence rates of OA by sex from 1990 to 2021. (A) Prevalence number and rate. (B) Incidence number and rate. Abbreviations: OA=osteoarthritis.
