# Supplementary material for: Global, regional, and national burden of osteoarthritis from 1990 to 2021 and projections to 2035: A cross-sectional study for the Global Burden of Disease Study 2021
Source: PLoS One. 2025 May 27;20(5):e0324296. doi: 10.1371/journal.pone.0324296 (PMC12111611; doi:10.1371/journal.pone.0324296)
Supplement: S2 Fig — Abbreviations: GBD = Global Burden of Disease. (DOCX) [file pone.0324296.s002.docx]

**S2 Fig. Contribution of different osteoarthritis sites to combined age-standardised prevalence, globally and by GBD region, 2021.**


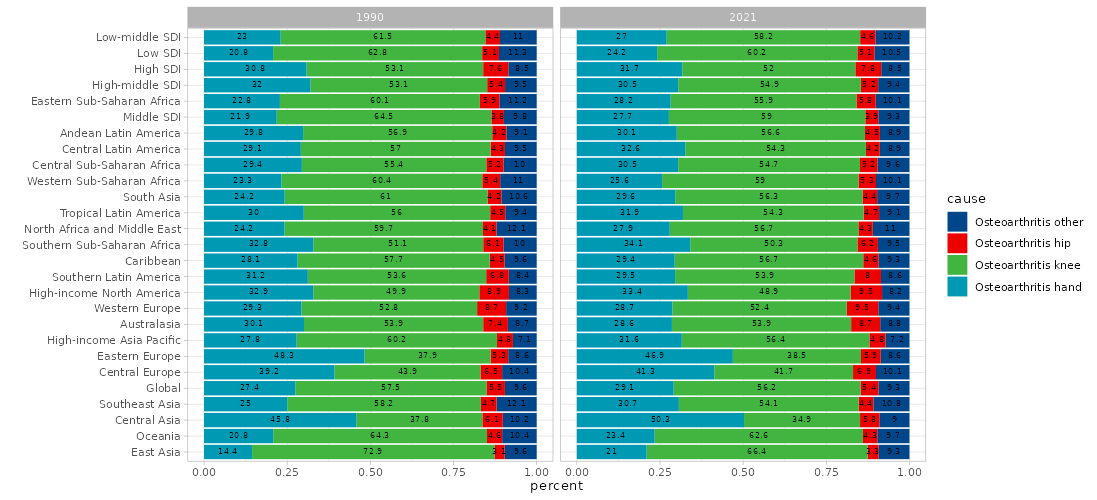


S2 Fig. Contribution of different osteoarthritis sites to combined age-standardised prevalence, globally and by GBD region, 2021. Abbreviations: GBD=Global Burden of Disease.
