# Supplementary material for: Global, regional, and national burden of osteoarthritis from 1990 to 2021 and projections to 2035: A cross-sectional study for the Global Burden of Disease Study 2021
Source: PLoS One. 2025 May 27;20(5):e0324296. doi: 10.1371/journal.pone.0324296 (PMC12111611; doi:10.1371/journal.pone.0324296)
Supplement: S3 Fig — Abbreviations: SDI = Socio-Demographic Index, OA = osteoarthritis, YLDs = years lived with disability. (DOCX) [file pone.0324296.s003.docx]

**S3 Fig. Frontier analysis based on SDI and OA YLDs in 204 countries and territories.**


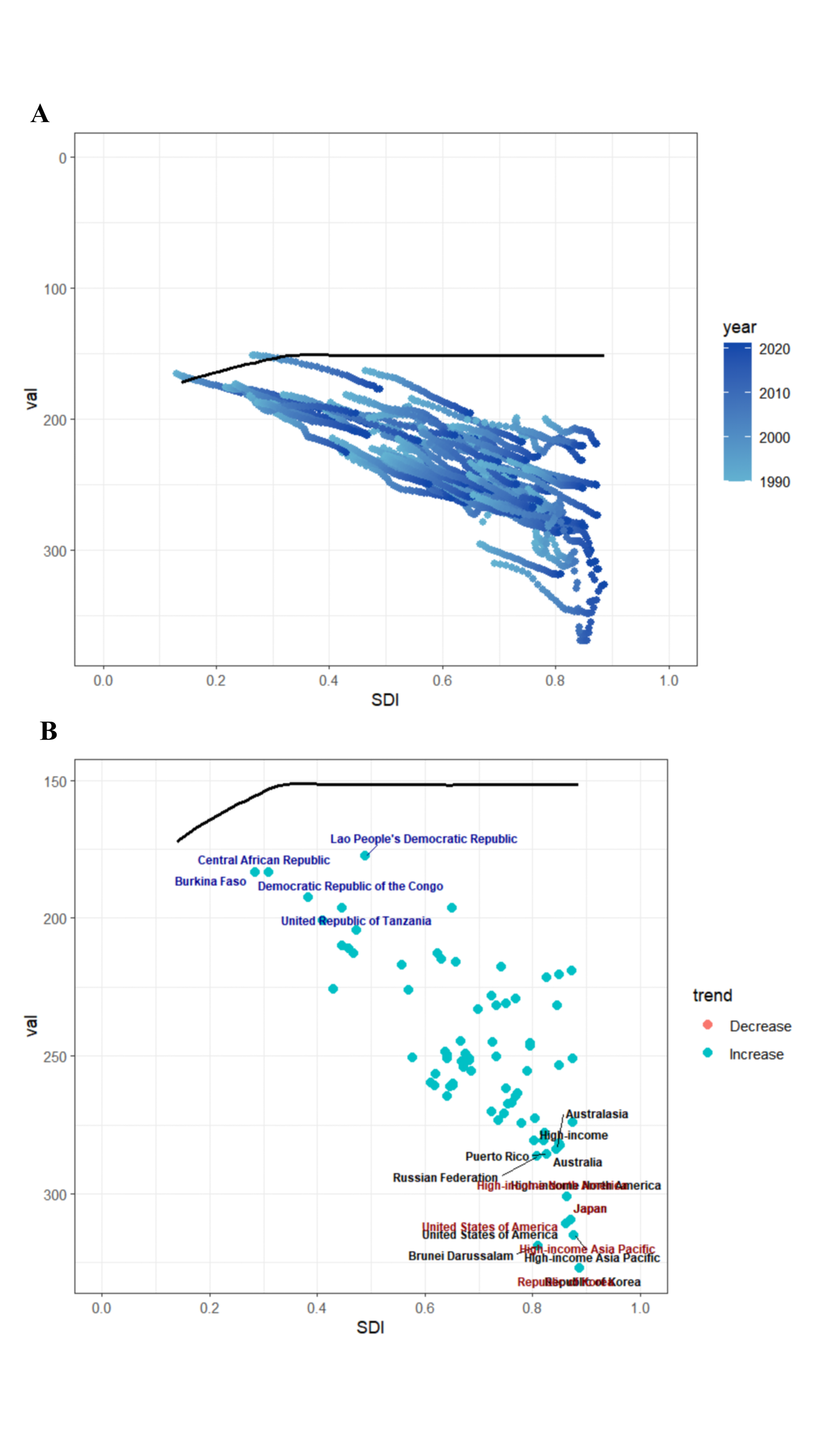


S3 Fig. Frontier analysis based on SDI and OA YLDs in 204 countries and territories. Abbreviations: SDI=Socio-Demographic Index, OA=osteoarthritis, YLDs=years lived with disability.
