# Supplementary material for: Global, regional, and national burden of osteoarthritis from 1990 to 2021 and projections to 2035: A cross-sectional study for the Global Burden of Disease Study 2021
Source: PLoS One. 2025 May 27;20(5):e0324296. doi: 10.1371/journal.pone.0324296 (PMC12111611; doi:10.1371/journal.pone.0324296)
Supplement: S4 Fig — Abbreviations: YLDs = years lived with disability, BMI = body mass index. (DOCX) [file pone.0324296.s004.docx]

**S4 Fig. The burden of age-standardised YLDs for osteoarthritis attributable to high BMI in 1990 vs 2021.**

**
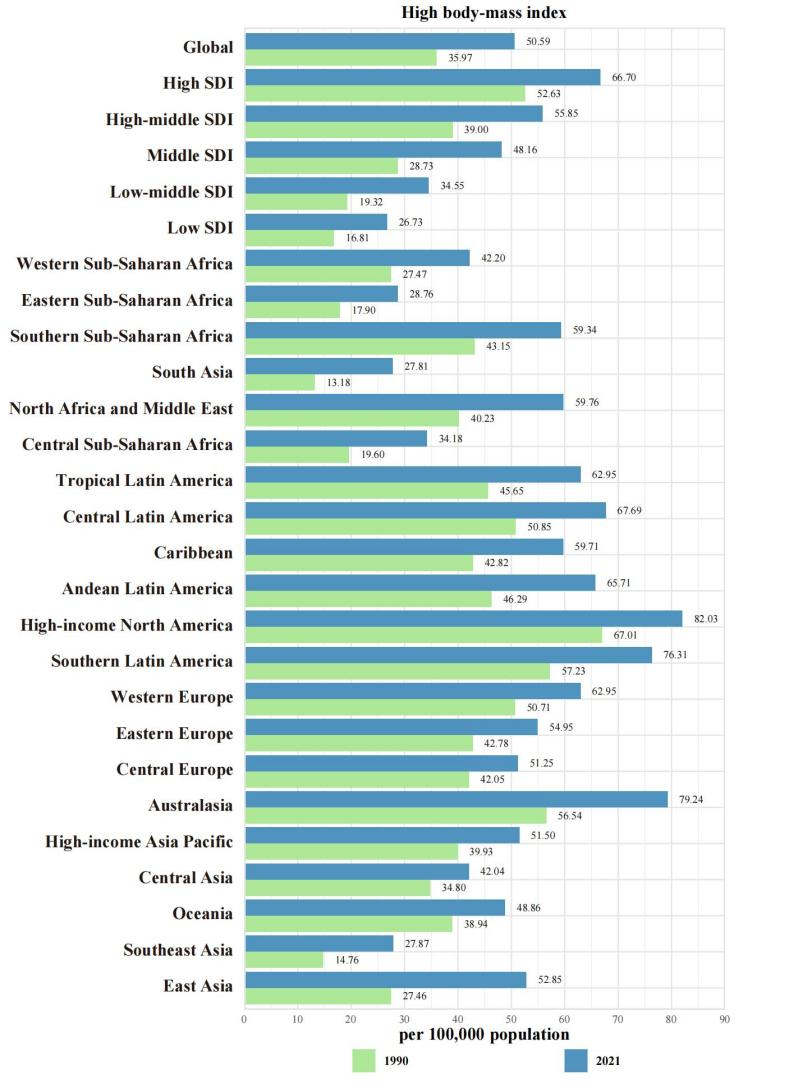
**

S4 Fig. The burden of age-standardised YLDs for osteoarthritis attributable to high BMI in 1990 vs 2021.Abbreviations: YLDs=years lived with disability, BMI=body mass index.
