# Supplementary material for: Global, regional, and national burden of osteoarthritis from 1990 to 2021 and projections to 2035: A cross-sectional study for the Global Burden of Disease Study 2021
Source: PLoS One. 2025 May 27;20(5):e0324296. doi: 10.1371/journal.pone.0324296 (PMC12111611; doi:10.1371/journal.pone.0324296)
Supplement: S1 Table — Abbreviations: YLDs = years lived with disability, OA = osteoarthritis, GBD = Global Burden of Disease. (DOCX) [file pone.0324296.s005.docx]

**S1 Table. The case number of prevalence, incidence and YLDs of OA in 1990 and 2021 for both sexes by GBD regions.**

Table1. The case number of prevalence, incidence and YLDs of OA in 1990 and 2021 for both sexes by GBD regions.

|  | 1990 | | | 2021 | | |
| --- | --- | --- | --- | --- | --- | --- |
|  | Number of YLDs | Number of Prevalence | Number of Incidence | Number of YLDs | Number of Prevalence | Number of Incidence |
| Global | 8918857 (4264151,17983776) | 256076700 (227119748,283438465) | 20900510 (18467653,23104316) | 21304566 (10189161,42935420) | 606989319 (537873608,670519617) | 46632144 (41122053,51644431) |
| East Asia | 1904132 (915864,3832084) | 55506569 (48473139,62109118) | 4836774 (4234495,5416117) | 5518037 (2633494,11061497) | 158285424 (139469183,176824968) | 12051700 (10562111,13556244) |
| Central Asia | 101371 (48802,204167) | 2890239 (2514610,3288610) | 221869 (194411,249130) | 212365 (101660,425622) | 6010310 (5207826,6870321) | 475854 (413121,535995) |
| Southeast Asia | 432058 (206865,871588) | 12662656 (11238788,14120228) | 1128950 (993293,1259580) | 1357628 (645150,2713214) | 39227935 (34567607,43609405) | 3261525 (2872405,3641133) |
| Oceania | 6038 (2899,12078) | 176696  (156978,197593) | 16659 (14676,18607) | 17457  (8450,34943) | 508189 (451273,565165) | 47743 (41835,53394) |
| Eastern Europe | 742363 (355456,1508177) | 21046221 (18447721,23746861) | 1495247 (1312342,1682319) | 965580 (465099,1951745) | 27107908 (23769327,30423620) | 1833038 (1612584,2058220) |
| Western Europe | 1327348 (642543,2667248) | 37369611 (33648956,41316152) | 2630868 (2349667,2928270) | 2131317 (1038440,4287323) | 59567389 (53848954,65960013) | 3918888 (3498382,4363204) |
| Southern Latin America | 115112 (54896,232022) | 3248110 (2899780,3599930) | 252630 (224153,281234) | 233580 (112227,468574) | 6538638 (5891362,7213429) | 483946 (431133,536648) |
| High-income North America | 964587 (463191,1948963) | 26834459 (24152314,29613959) | 1899645 (1695714,2093720) | 1857796 (900403,3761748) | 51749679 (46318473,57318852) | 3457087 (3062826,3850766) |
| Caribbean | 59541  (28499,120118) | 1698190 (1502561,1877189) | 139108 (123461,154387) | 135591 (64908,274656) | 3852892 (3412425,4252980) | 296170 (262547,330668) |
| Andean Latin America | 48494  (23257,97370) | 1383606 (1228226,1528853) | 121137 (107214,134640) | 156693 (75061,316245) | 4431105 (3933346,4885804) | 363396 (319748,402547) |
| Central Latin America | 197933 (94851,399110) | 5697761 (5042112,6304605) | 509648 (450946,568026) | 676745 (322649,1368410) | 19197614 (16954459,21143360) | 1561605 (1379181,1731311) |
| Tropical Latin America | 213399 (102015,428853) | 6184012 (5480738,6851449) | 551639 (485844,614416) | 678602 (325615,1368810) | 19391655 (17180012,21545741) | 1568533 (1385103,1733206) |
| North Africa and Middle East | 319580 (153014,643843) | 9337845 (8286676,10395164) | 839234 (738099,934460) | 1049857 (503111,2115549) | 30491685 (27064615,33709288) | 2730761 (2402789,3044967) |
| Central Europe | 327001 (157196,659825) | 9379903 (8274711,10510034) | 699280 (618328,779466) | 514447 (248117,1044284) | 14519446 (12794343,16234191) | 960144 (849389,1070329) |
| Australasia | 58807 (28504,118543) | 1659800 (1490005,1834682) | 124186 (110781,137553) | 140906 (69472,286856) | 3922514 (3537651,4335715) | 269452 (239374,301169) |
| South Asia | 1094410 (528656,2197896) | 32454745 (28765905,35895035) | 2993308 (2640026,3326476) | 3311236 (1583934,6656919) | 96531169 (85576494,106691001) | 8220378 (7241991,9115916) |
| High-income Asia Pacific | 597447 (286964,1207310) | 16572952 (14690574,18303601) | 1345776 (1189858,1491373) | 1276815 (611789,2579654) | 34625939 (31148250,37978128) | 2189403 (1958142,2413152) |
| Central Sub-Saharan Africa | 44741  (21342,90400) | 1314105 (1159131,1464371) | 122007 (106783,135467) | 120682 (57012,241714) | 3517438 (3115894,3914394) | 339701 (298431,378043) |
| Eastern Sub-Saharan Africa | 132919 (63595,267611) | 3912597 (3470701,4378272) | 366643 (322665,409382) | 358151 (171684,719704) | 10409572 (9265846,11557319) | 1000971 (885084,1115451) |
| Southern Sub-Saharan Africa | 62841 (30077,125999) | 1801904 (1590783,2000266) | 157577 (139016,175112) | 148991 (71571,298108) | 4289179 (3773086,4755346) | 375228 (330227,417302) |
| Western Sub-Saharan Africa | 168735 (81206,339506) | 4944718 (4378452,5533517) | 448326 (395308,501301) | 442087 (212439,888884) | 12813642 (11400709,14172790) | 1226622 (1078017,1368997) |

Abbreviations: YLDs=years lived with disability, ASR=Age-standardised rate, OA=osteoarthritis, GBD=Global Burden of Disease.
