# Supplementary material for: Global, regional, and national burden of osteoarthritis from 1990 to 2021 and projections to 2035: A cross-sectional study for the Global Burden of Disease Study 2021
Source: PLoS One. 2025 May 27;20(5):e0324296. doi: 10.1371/journal.pone.0324296 (PMC12111611; doi:10.1371/journal.pone.0324296)
Supplement: S2 Table — Abbreviations: OA = osteoarthritis. (DOCX) [file pone.0324296.s006.docx]

**S2 Table. Joinpoint regression analysis of the sex-specific age-standardized incidence rate for OA globally from 1990 to 2021.**

**2.1 Age-standardized incidence rate for both sexes**

| **Estimated Joinpoints** | | | | | | | | | | | | | | | | |  |  | |  | | |  |  |  |  | | |  |
| --- | --- | --- | --- | --- | --- | --- | --- | --- | --- | --- | --- | --- | --- | --- | --- | --- | --- | --- | --- | --- | --- | --- | --- | --- | --- | --- | --- | --- | --- |
| **Cohort** | | | **Joinpoint** | | | | | **Estimate** | | | **Lower CI** | | | | **Upper CI** | |  |  | |  | | |  |  |  |  | | |  |
| Both | | | 1 | | | | | 1994 | | | 1992 | | | | 2002 | |  |  | |  | | |  |  |  |  | | |  |
| Both | | | 2 | | | | | 2000 | | | 1995 | | | | 2006 | |  |  | |  | | |  |  |  |  | | |  |
| Both | | | 3 | | | | | 2005 | | | 1999 | | | | 2010 | |  |  | |  | | |  |  |  |  | | |  |
| Both | | | 4 | | | | | 2009 | | | 2004 | | | | 2016 | |  |  | |  | | |  |  |  |  | | |  |
| Both | | | 5 | | | | | 2015 | | | 2012 | | | | 2019 | |  |  | |  | | |  |  |  |  | | |  |
|  |  |  |  |  |  |  |  |  |  |  |  |  |  | |  |  |  | |  | |  |  | |  |  |  |  |  | |
| **Annual Percent Change (APC)** | | | | | | | | | | | | | | | | | | | | | | | | | | | | |  |
| **Cohort** | | **Segment** | | | | **Lower EndPoint** | | | | **Upper Endpoint** | | | **APC** | | | **Lower CI** | | | | **Upper CI** | | | **Test Statistic (t)** | | | **Prob > \|t\|** | | |  |
| Both | | 1 | | | | 1990 | | | | 1994 | | | 0.1533* | | | 0.1253 | | | | 0.1813 | | | 11.6772 | | | < 0.000001 | | |  |
| Both | | 2 | | | | 1994 | | | | 2000 | | | 0.0851* | | | 0.0655 | | | | 0.1047 | | | 9.2669 | | | < 0.000001 | | |  |
| Both | | 3 | | | | 2000 | | | | 2005 | | | 0.5468* | | | 0.5191 | | | | 0.5745 | | | 42.2170 | | | < 0.000001 | | |  |
| Both | | 4 | | | | 2005 | | | | 2009 | | | 0.4201* | | | 0.3771 | | | | 0.4631 | | | 20.8555 | | | < 0.000001 | | |  |
| Both | | 5 | | | | 2009 | | | | 2015 | | | 0.3334* | | | 0.3144 | | | | 0.3525 | | | 37.3690 | | | < 0.000001 | | |  |
| Both | | 6 | | | | 2015 | | | | 2021 | | | 0.2249* | | | 0.2100 | | | | 0.2398 | | | 32.2577 | | | < 0.000001 | | |  |
| * Indicates that the Annual Percent Change (APC) is significantly different from zero at the alpha = 0.05 level. | | | | | | | | | | | | | | | | | | | | | | | | | | | | |  |
| **Average Annual Percent Change (AAPC)** | | | | | | | | | | | | | | | | | | | | | | | | | | | | |  |
| **Cohort** | | **Range** | | | | | **Lower EndPoint** | | **Upper Endpoint** | | | **AAPC** | | **Lower CI** | | | | **Upper CI** | | | | | **Test Statistic ~** | | | **P-Value ~** | | |  |
| Both | | Full Range | | | | | 1990 | | 2021 | | | 0.2866* | | 0.2774 | | | | 0.2958 | | | | | 61.1847 | | | < 0.000001 | | |  |
| * Indicates that the AAPC is significantly different from zero at the alpha = 0.05 level. ~ If the AAPC is within one segment, the t-distribution is used. Otherwise, the normal (z) distribution is used. | | | | | | | | | | | | | | | | | | | | | | | | | | | | |  |

CI: confidence interval.

**2.2 Age-standardized incidence rate for male**

| **Estimated Joinpoints** | | | | | | | | | | | | | | | | |  |  | |  | | |  |  |  |  | | |  |
| --- | --- | --- | --- | --- | --- | --- | --- | --- | --- | --- | --- | --- | --- | --- | --- | --- | --- | --- | --- | --- | --- | --- | --- | --- | --- | --- | --- | --- | --- |
| **Cohort** | | | **Joinpoint** | | | | | **Estimate** | | | **Lower CI** | | | | **Upper CI** | |  |  | |  | | |  |  |  |  | | |  |
| Male | | | 1 | | | | | 2000 | | | 1999 | | | | 2001 | |  |  | |  | | |  |  |  |  | | |  |
| Male | | | 2 | | | | | 2005 | | | 2004 | | | | 2006 | |  |  | |  | | |  |  |  |  | | |  |
| Male | | | 3 | | | | | 2010 | | | 2009 | | | | 2011 | |  |  | |  | | |  |  |  |  | | |  |
| Male | | | 4 | | | | | 2016 | | | 2013 | | | | 2016 | |  |  | |  | | |  |  |  |  | | |  |
| Male | | | 5 | | | | | 2019 | | | 2017 | | | | 2019 | |  |  | |  | | |  |  |  |  | | |  |
|  |  |  |  |  |  |  |  |  |  |  |  |  |  | |  |  |  | |  | |  |  | |  |  |  |  |  | |
| **Annual Percent Change (APC)** | | | | | | | | | | | | | | | | | | | | | | | | | | | | |  |
| **Cohort** | | **Segment** | | | | **Lower EndPoint** | | | | **Upper Endpoint** | | | **APC** | | | **Lower CI** | | | | **Upper CI** | | | **Test Statistic (t)** | | | **Prob > \|t\|** | | |  |
| Male | | 1 | | | | 1990 | | | | 2000 | | | 0.1231* | | | 0.1169 | | | | 0.1293 | | | 42.4880 | | | < 0.000001 | | |  |
| Male | | 2 | | | | 2000 | | | | 2005 | | | 0.5603* | | | 0.5353 | | | | 0.5853 | | | 47.9224 | | | < 0.000001 | | |  |
| Male | | 3 | | | | 2005 | | | | 2010 | | | -0.0850* | | | -0.1093 | | | | -0.0606 | | | -7.4418 | | | 0.000002 | | |  |
| Male | | 4 | | | | 2010 | | | | 2016 | | | 0.4505* | | | 0.4335 | | | | 0.4675 | | | 56.6696 | | | < 0.000001 | | |  |
| Male | | 5 | | | | 2016 | | | | 2019 | | | 0.6453* | | | 0.5669 | | | | 0.7238 | | | 17.5883 | | | < 0.000001 | | |  |
| Male | | 6 | | | | 2019 | | | | 2021 | | | 0.2543* | | | 0.1746 | | | | 0.3342 | | | 6.8009 | | | 0.000006 | | |  |
| * Indicates that the Annual Percent Change (APC) is significantly different from zero at the alpha = 0.05 level. | | | | | | | | | | | | | | | | | | | | | | | | | | | | |  |
|  |  |  |  |  |  |  |  |  |  |  |  |  |  | |  |  |  | |  | |  |  | |  |  |  |  |  | |
| **Average Annual Percent Change (AAPC)** | | | | | | | | | | | | | | | | | | | | | | | | | | | | |  |
| **Cohort** | | **Range** | | | | | **Lower EndPoint** | | **Upper Endpoint** | | | **AAPC** | | **Lower CI** | | | | **Upper CI** | | | | | **Test Statistic ~** | | | **P-Value ~** | | |  |
| Male | | Full Range | | | | | 1990 | | 2021 | | | 0.2821* | | 0.2716 | | | | 0.2926 | | | | | 52.7798 | | | < 0.000001 | | |  |
| * Indicates that the AAPC is significantly different from zero at the alpha = 0.05 level. ~ If the AAPC is within one segment, the t-distribution is used. Otherwise, the normal (z) distribution is used. | | | | | | | | | | | | | | | | | | | | | | | | | | | | |  |

CI: confidence interval.

**2.3 Age-standardized incidence rate for female**

| **Estimated Joinpoints** | | | | | | | | | | | | | | | | |  |  | |  | | |  |  |  |  | | |  |
| --- | --- | --- | --- | --- | --- | --- | --- | --- | --- | --- | --- | --- | --- | --- | --- | --- | --- | --- | --- | --- | --- | --- | --- | --- | --- | --- | --- | --- | --- |
| **Cohort** | | | **Joinpoint** | | | | | **Estimate** | | | **Lower CI** | | | | **Upper CI** | |  |  | |  | | |  |  |  |  | | |  |
| Female | | | 1 | | | | | 1994 | | | 1992 | | | | 2002 | |  |  | |  | | |  |  |  |  | | |  |
| Female | | | 2 | | | | | 2000 | | | 1999 | | | | 2007 | |  |  | |  | | |  |  |  |  | | |  |
| Female | | | 3 | | | | | 2006 | | | 2003 | | | | 2011 | |  |  | |  | | |  |  |  |  | | |  |
| Female | | | 4 | | | | | 2009 | | | 2008 | | | | 2016 | |  |  | |  | | |  |  |  |  | | |  |
| Female | | | 5 | | | | | 2014 | | | 2012 | | | | 2019 | |  |  | |  | | |  |  |  |  | | |  |
|  |  |  |  |  |  |  |  |  |  |  |  |  |  | |  |  |  | |  | |  |  | |  |  |  |  |  | |
| **Annual Percent Change (APC)** | | | | | | | | | | | | | | | | | | | | | | | | | | | | |  |
| **Cohort** | | **Segment** | | | | **Lower EndPoint** | | | | **Upper Endpoint** | | | **APC** | | | **Lower CI** | | | | **Upper CI** | | | **Test Statistic (t)** | | | **Prob > \|t\|** | | |  |
| Female | | 1 | | | | 1990 | | | | 1994 | | | 0.1868* | | | 0.1110 | | | | 0.2626 | | | 5.2559 | | | 0.000097 | | |  |
| Female | | 2 | | | | 1994 | | | | 2000 | | | 0.0586* | | | 0.0056 | | | | 0.1117 | | | 2.3554 | | | 0.032535 | | |  |
| Female | | 3 | | | | 2000 | | | | 2006 | | | 0.5455* | | | 0.4924 | | | | 0.5985 | | | 21.9776 | | | < 0.000001 | | |  |
| Female | | 4 | | | | 2006 | | | | 2009 | | | 0.8732* | | | 0.6406 | | | | 1.1064 | | | 8.0267 | | | 0.000001 | | |  |
| Female | | 5 | | | | 2009 | | | | 2014 | | | 0.3159* | | | 0.2434 | | | | 0.3885 | | | 9.2961 | | | < 0.000001 | | |  |
| Female | | 6 | | | | 2014 | | | | 2021 | | | 0.0313 | | | -0.0003 | | | | 0.0629 | | | 2.1142 | | | 0.051654 | | |  |
| * Indicates that the Annual Percent Change (APC) is significantly different from zero at the alpha = 0.05 level. | | | | | | | | | | | | | | | | | | | | | | | | | | | | |  |
|  |  |  |  |  |  |  |  |  |  |  |  |  |  | |  |  |  | |  | |  |  | |  |  |  |  |  | |
| **Average Annual Percent Change (AAPC)** | | | | | | | | | | | | | | | | | | | | | | | | | | | | |  |
| **Cohort** | | **Range** | | | | | **Lower EndPoint** | | **Upper Endpoint** | | | **AAPC** | | **Lower CI** | | | | **Upper CI** | | | | | **Test Statistic ~** | | | **P-Value ~** | | |  |
| Female | | Full Range | | | | | 1990 | | 2021 | | | 0.2832* | | 0.2542 | | | | 0.3122 | | | | | 19.1471 | | | < 0.000001 | | |  |
| * Indicates that the AAPC is significantly different from zero at the alpha = 0.05 level. ~ If the AAPC is within one segment, the t-distribution is used. Otherwise, the normal (z) distribution is used. | | | | | | | | | | | | | | | | | | | | | | | | | | | | |  |

CI: confidence interval.
