# Supplementary material for: Global, regional, and national burden of osteoarthritis from 1990 to 2021 and projections to 2035: A cross-sectional study for the Global Burden of Disease Study 2021
Source: PLoS One. 2025 May 27;20(5):e0324296. doi: 10.1371/journal.pone.0324296 (PMC12111611; doi:10.1371/journal.pone.0324296)
Supplement: Attachment — Submitted filename: Review.docx [file pone.0324296.s007.docx]

Thank you for the opportunity to review this manuscript.

The study addresses a crucial topic regarding the prevalence and burden of osteoarthritis (OA) on a global scale. Given the increasing impact of OA on individuals and healthcare systems, it is vital to have up-to-date prevalence estimates and projections for effective public health planning.

However, a study published in 2023 already presents the burden of OA and its projections based on data from GBD 2021, which raises some doubts regarding the relevance of this study.

Nonetheless, I provide below several comments and suggestions that I believe could enhance the clarity, rigor, and overall quality of the manuscript

#2 / Line 3

Please provide the full term before using the abbreviation SDI.

#3

The conclusion in the abstract seems too long and repetitive with the results. I suggest that the authors focus on the results in the previous section and leave one or two paragraphs to conclude with the overall burden of OA and its implications for health policies

#4 Lines 2-5

I suggest to use a full stop after “populations” and to combine the sentences where the authors describe the pathological changes and the clinic manifestations.

#4 Lines 6-7

The statement '… making OA one of the leading causes of chronic pain and long-term disability in older adults' requires a citation.

#4 Lines 8-9

The statement “With the rapid acceleration of global aging and the sharp rise in obesity rates across all age groups, the incidence of OA continues to increase” requires a citation.

Additionally, it would be important to previously state the role of age and obesity as risk factors for the development of OA, in order to clarify the following idea for non-expert readers

#4 Lines 9-10

In the sentence “	As of 2020, the global prevalence of OA had risen by 132.2% compared to 1990”, I would suggest to use “By” instead of “As of”. Additionally, the sentence requires a citation.

\# 4 Lines 27-28

As mentioned earlier, I believe this is not the first study to estimate the burden of OA across all global regions based on GBD 2021 data. If the authors believe that this study differs from the one presented in this article (https://pmc.ncbi.nlm.nih.gov/articles/PMC10477960/pdf/main.pdf), they should acknowledge the existence of the previous study and highlight the differences, as well as what new insights their study brings.

Line 29

Please provide the full term before using the abbreviation ASR.

 STUDY DESIGN AND METHODS

* Data Sources and Methods

It would be important to clarify what OA sites are being considered in the study

#5 Lines 13-15

In order to clarify the idea stated in the sentence 'DALY is the standardized measure used to quantify the burden of disease. As the cause-of-death model of GBD assumes no deaths attributable to OA, the DALY for OA is equivalent to YLD,' it would be important to previously explain that DALY = YLL + YLD."

#5 Lines 20-21

I was surprised by the use of a reporting guideline specifically designed for surgical studies, when other guidelines for reporting observational studies, such as STROBE, are available. Can you please clarify this option?

*  Case Definition

The authors describe the case definition for knee and hip OA, but not for hand or other sites OA

#7 Line 4

Please remove one “a” before “country’s”

#14 Lines 19-22

Considering that the authors listed factors such as alcohol consumption, smoking, low calcium intake, low physical activity levels, high blood pressure, high blood glucose, and high BMI, it would be helpful to clarify which of these factors are included under 'metabolic risk.

Additionally, the authors should justify the inclusion of these factors. The selection of risk factors should be based on clear criteria, such as, for example, epidemiological evidence of causality.

#19 Lines 9 - 30

First, I would suggest that the authors reformulate all the paragraph, using collons after each strategie.

Nonetheless, I have some doubts about the need for the authors to provide specific proposals in each strategy. For example, in the 'Weight Management and Healthy Diet Education' strategy, the statement 'A balanced diet should include a variety of vegetables, fruits, whole grains, low-fat proteins, and foods rich in omega-3 fatty acids (e.g., fish and nuts) to alleviate the burden of weight on the joints' might be too detailed. I believe this is not the focus of the study, and the authors should limit their discussion to presenting the general strategies that should be implemented, leaving the specification of interventions to other sources

#20 Lines 1-9

The same as before. I believe that the authors should refrain from this type of analysis, which, in my opinion, deviates from the focus of the study.

I believe the discussion would be enhanced with some suggestions for future research. The authors address the topic when describing the need to study more risk factors, but they do not define clear lines of research needs. ch needs.
