# Supplementary material for: Global, regional, and national burden of osteoarthritis from 1990 to 2021 and projections to 2035: A cross-sectional study for the Global Burden of Disease Study 2021
Source: PLoS One. 2025 May 27;20(5):e0324296. doi: 10.1371/journal.pone.0324296 (PMC12111611; doi:10.1371/journal.pone.0324296)
Supplement: Attachment — Submitted filename: Response to Reviewers.docx [file pone.0324296.s009.docx]

**Response to Reviewers – Point-by-Point Reply**

Dear Editor and Reviewers,

Thank you for taking the time to review our manuscript and providing valuable comments and suggestions. On behalf of all the authors, I would like to express our sincere gratitude for your insightful feedback. After thorough discussions among the co-authors, we have addressed your suggestions and made revisions to the manuscript accordingly. The modified sections have been highlighted using the track changes mode. Below, we provide a point-by-point response to the reviewers' comments:

**Reviewer #1: The study addresses a crucial topic regarding the prevalence and burden of osteoarthritis (OA) on a global scale. Given the increasing impact of OA on individuals and healthcare systems, it is vital to have up-to-date prevalence estimates and projections for effective public health planning.**

**However, a study published in 2023 already presents the burden of OA and its projections based on data from GBD 2021, which raises some doubts regarding the relevance of this study.**

**Nonetheless, I provide below several comments and suggestions that I believe could enhance the clarity, rigor, and overall quality of the manuscript.**

**Response:** Thank you very much for your valuable insights. We fully understand and acknowledge your concerns. The issue you raised was also a key consideration during the initial design of this study. We had previously recognized that without restricting factors such as region and population, the estimated burden of osteoarthritis (OA) might closely resemble the findings from prior analyses using data from the Global Burden of Disease study. However, considering the widespread impact of the COVID-19 pandemic since 2019 ^[1]^, along with the accelerating population aging trends in major global economies, particularly in the Asia-Pacific region, we hypothesize that the prevalence of OA may be undergoing subtle changes.**We therefore adopted a more diversified approach to analyze the global burden of OA from different perspectives, such as time, region, and gender, which led to some completely new conclusions.**

As confirmed in our study, the projected age-standardized prevalence rate (ASPR) of OA over the next 15 years does not show a continuous increase, as previously assumed. Instead, it demonstrates an overall declining trend. This might be attributable to advancements in OA prevention and treatment, as well as strengthened international medical collaboration ^[2]^. Furthermore, the GBD team has also emphasized the necessity of conducting epidemiological analyses using GBD 2021 data ^[3]^.

Compared to previously published studies on global OA epidemiology, **this study employed methods such as trend analysis, decomposition analysis, frontier analysis, and predictive analysis to examine the burden of OA from multiple perspectives, including gender, age, and time.** First, we observed that **the ASPR of OA was highest in the high-income Asia-Pacific region rather than in high-income North America**, as previously reported. At the national level, **the highest ASPR shifted from the United States to South Korea**, with countries such as Singapore, Brunei, and Japan also bearing a significant OA burden, indicating a notable geographic shift in OA burden. Second, our frontier analysis revealed that **high-SDI (Socio-Demographic Index) countries, including the United States, Japan, and South Korea, bear a disproportionately high OA burden**. Based on this information, we updated the global status of OA.

Additionally, we conducted a further analysis of OA risk factors. We found that, compared to GBD 2019, which included only BMI as a risk factor, **GBD 2021 incorporates two major risk factors: metabolic risks and high BMI**. This provides an updated and more comprehensive reference for future research ^[4]^.

**Reviewer #1: Please provide the full term before using the abbreviation SDI.**

**Response:** Thanks to your careful attention, we have refined the full name of the SDI - Socio-Demographic Index - and correctly labeled the first occurrence of “SDI” in the text.

**Reviewer #1: The conclusion in the abstract seems too long and repetitive with the results. I suggest that the authors focus on the results in the previous section and leave one or two paragraphs to conclude with the overall burden of OA and its implications for health policies.**

**Response:**We have shortened the word count of our conclusions and followed your suggestion to elaborate on our findings on the global burden of OA in the results section. In addition, in the conclusions section, we summarize **the overall burden of OA, the current challenges, and what strategies the public health sector sector should adopt to address these changes**.

**Reviewer #1: I suggest to use a full stop after “populations” and to combine the sentences where the authors describe the pathological changes and the clinic manifestations.**

**Response:**Thank you for your suggestion, our previous presentation was indeed not fluent enough, we have broken the sentences as you suggested and reintegrated the discourse on the pathological changes and clinical manifestations of osteoarthritis.

**Reviewer #1: The statement '… making OA one of the leading causes of chronic pain and long-term disability in older adults' requires a citation.**

**Response:**Thank you for the reminder that references do need to be added here to further support the formulation, and we have already cited the quote.

**Reviewer #1: The statement “With the rapid acceleration of global aging and the sharp rise in obesity rates across all age groups, the incidence of OA continues to increase” requires a citation.Additionally, it would be important to previously state the role of age and obesity as risk factors for the development of OA, in order to clarify the following idea for non-expert readers.**

**Response:**Thank you for the reminder that your suggestion does have an important role to play for a wider potential audience, and we have quoted the quote, followed by a brief elaboration on **how age and obesity as risk factors affect osteoarthritis**.

**Reviewer #1: In the sentence “	As of 2020, the global prevalence of OA had risen by 132.2% compared to 1990”, I would suggest to use “By” instead of “As of”. Additionally, the sentence requires a citation.**

**Response:**Thanks to your careful reminders, we have revised and improved the grammar and wording as you suggested, and we have quoted the sentence.

**Reviewer #1: As mentioned earlier, I believe this is not the first study to estimate the burden of OA across all global regions based on GBD 2021 data. If the authors believe that this study differs from the one presented in this article (https://pmc.ncbi.nlm.nih.gov/articles/PMC10477960/pdf/main.pdf), they should acknowledge the existence of the previous study and highlight the differences, as well as what new insights their study brings.**

**Response:**Thanks to your suggestion, we recognize the contribution of this article (https://pmc.ncbi.nlm.nih.gov/articles/PMC10477960/pdf/main.pdf). It is important to emphasize how our study differs from previous studies, and therefore we have enhanced the relevant statements in the introduction section to highlight the differences in this study and in the discussion section to emphasize the new insights we have presented.

Admittedly, this article has provided a more comprehensive analysis of the disease burden of OA, but it is important to note that our article still has many unique features. First, this article, published in Lancet Rheumatol, used three main approaches, **namely descriptive, decomposition, and predictive analyses**, to illustrate the global burden of OA and regional differences. Our article, on the other hand, **used a more diversified approach and drew some new conclusions**. First, we described **the temporal trends of OA burden**, **regional distribution changes**, and **the changes of OA burden over time through joinpoint analysis**; second, we also **described the gender differences of OA burden**, and we found that women have a heavier burden of OA than men (in terms of incidence and prevalence); in addition to this, we found that within the framework of GBD 2021, **BMI and metabolic factors are the OA two major risk factors**, which is a major step forward compared with the previous approach of using BMI only as a risk factor for OA; finally, we applied the method of **frontier analysis** to analyze the differences in the expected effects of combating OA in different SDI regions. In conclusion, our article has similarities with this one, but **we adopted a more multifaceted approach to analyze the disease burden of OA and derived some brand new insights**, which we believe will help us to better understand how OA has changed over the past 30 years and how we should respond to the new challenges posed by OA under the conditions of the new era.

**Reviewer #1: Please provide the full term before using the abbreviation ASR.**

**Response:**Thanks to your reminder, we have refined the full name of the ASR - age-standardized rate - and correctly marked the first occurrence of “ASR” in the text.

**Reviewer #1: It would be important to clarify what OA sites are being considered in the study.**

**Response:**We extracted OA data for GBD 2021 including: total OA, knee osteoarthritis, hip osteoarthritis, hand osteoarthritis and other osteoarthritis. We added in **2.1 Data Sources and Methods** section about the OA sites covered in this paper.

**Reviewer #1: In order to clarify the idea stated in the sentence 'DALY is the standardized measure used to quantify the burden of disease. As the cause-of-death model of GBD assumes no deaths attributable to OA, the DALY for OA is equivalent to YLD,' it would be important to previously explain that DALY = YLL + YLD."**

**Response:**Your suggestion is very pertinent and we have added the relationship “DALY = YLL + YLD” to the original text so that potential readers can then better understand the OA burden.

**Reviewer #1: I was surprised by the use of a reporting guideline specifically designed for surgical studies, when other guidelines for reporting observational studies, such as STROBE, are available. Can you please clarify this option?**

**Response:**In practice, there is no generalized specification for data use of the GBD, and the guideline STROCSS 2024 guidelines was chosen because we found that there is reliable paper (https://journals.lww.com/international-journal-of-surgery/ fulltext/2025/02000/the_global,_regional,_and_national_burden_of.9.aspx) used this specification. However, after your reminder, we have reorganized the structure of this paper and carefully read the specifications of the **GATHER** declaration, the STROBE declaration, and so on, and after careful consideration we believe that this paper should follow the **STROBE** declaration. Therefore, we explain the specifications that should be followed in this paper in section **2.1 Data Sources and Methods**.

**Reviewer #1: The authors describe the case definition for knee and hip OA, but not for hand or other sites OA.**

**Response:**We added a definition of hand and other sites OA in the **2.3 Case Definition** section.

**Reviewer #1: Please remove one “a” before “country’s”.**

**Response:**We appreciate your careful review and have removed inappropriate words based on your suggestions.

**Reviewer #1: Considering that the authors listed factors such as alcohol consumption, smoking, low calcium intake, low physical activity levels, high blood pressure, high blood glucose, and high BMI, it would be helpful to clarify which of these factors are included under 'metabolic risk.**

**Additionally, the authors should justify the inclusion of these factors. The selection of risk factors should be based on clear criteria, such as, for example, epidemiological evidence of causality.**

**Response:Within the GBD framework, metabolic risk includes: high blood glucose, high blood pressure, low bone density, high LDL cholesterol, high BMI, and impaired kidney function**. After we included all the above factors in our analysis, according to the output of GBD (https://vizhub.healthdata.org/gbd-results/): high BMI and total metabolic risk were the only two risk factors. These factors were chosen because: **high blood glucose, which may cause accumulation of glycosylation end products leading to inflammation and cartilage damage; high blood pressure, which may be associated with intra-articular vasculopathy, affects cartilage nutrient supply; low bone mineral density, which may alter the biomechanics of the joints and increase the cartilage stress; high LDL cholesterol, which may promote inflammation and oxidative stress; high BMI, which increases the mechanical load on joints with the concomitant release of adipose tissue inflammatory factors; impaired renal function may affect calcium and phosphorus metabolism, leading to abnormal cartilage mineralization.** Dong^[5]^ also showed that the cohort with High BMI and Inflammation-Related had the highest risk of osteoarthritis and death, which was associated with obesity-related genetic variants. We elaborate on what metabolic risk includes and why we chose these factors as risk factors for OA in section **3.4 Risk Factor Analysis for OA**.

**Reviewer #1: First, I would suggest that the authors reformulate all the paragraph, using collons after each strategie.**

**Nonetheless, I have some doubts about the need for the authors to provide specific proposals in each strategy. For example, in the 'Weight Management and Healthy Diet Education' strategy, the statement 'A balanced diet should include a variety of vegetables, fruits, whole grains, low-fat proteins, and foods rich in omega-3 fatty acids (e.g., fish and nuts) to alleviate the burden of weight on the joints' might be too detailed. I believe this is not the focus of the study, and the authors should limit their discussion to presenting the general strategies that should be implemented, leaving the specification of interventions to other sources.**

**Response:**Your suggestion is very true. We have re-edited the paragraphs and used colons to separate them so that they look cleaner and flow better. We have also removed redundancies and clutter, so that the interventions are presented in a more concise manner and the article is more focused on the topic.

**Reviewer #1: The same as before. I believe that the authors should refrain from this type of analysis, which, in my opinion, deviates from the focus of the study.**

**I believe the discussion would be enhanced with some suggestions for future research. The authors address the topic when describing the need to study more risk factors, but they do not define clear lines of research needs.**

**Response:**We have removed the discussion of the mechanism of analgesic action of NSAIDs, thus making the article more relevant to the focus of the study. **Suggestions for future research are also stated in the concluding part of the discussion.**

The specific revisions are as follows:

**Abstract:**

***(Page 3 Line 14-22)*** **Conclusions:**.........“As a major public health problem, the overall global burden of OA has shown an upward trend from 1990 to 2019, including an increase in the number of cases and inequalities in distribution across the globe, which has resulted in significant health losses and economic burdens. In addition, SDI-related inequalities between countries are increasing. In this regard, national public health authorities and the World Health Organization (WHO) should work together to improve diagnosis and early treatment rates by strengthening disease awareness and education, as well as strengthening international cooperation, providing necessary medical assistance to less developed regions, and actively exploring new strategies for the prevention and treatment of OA.”

**Introduction:**

***(Page 5 Line 1-6)*** **Introduction:**..........“Osteoarthritis (OA) is the most common musculoskeletal disorder among middle-aged and elderly populations. It is characterized by pathological changes such as cartilage degeneration, bone remodeling, and osteophyte formation, and its clinical manifestations include joint pain, stiffness, swelling, and functional limitations.”

***(Page 5 Line 8-16)*** **Introduction:**..........“With the rapid acceleration of global aging and the sharp rise in obesity rates across all age groups, the incidence of OA continues to increase, due to the fact that with age, **articular cartilage undergoes progressive degeneration due to prolonged mechanical loading and diminished self-repairing ability**, which directly increases the risk of OA development; at the same time, **obesity exerts excessive mechanical stress on weight-bearing joints (especially the knees), and the adipose tissue secretes pro-inflammatory cytokines that exacerbate cartilage degeneration and synovial inflammation**. **By 2020**, the global prevalence of OA had risen by 132.2% compared to 1990.”

***(Page 6 Line 10-17)*** **Introduction:**..........“To the best of our knowledge, this study is the first to estimate the results of GBD 2021 using multivariate methods to analyze the burden of OA in all regions of the world. We used **descriptive, trend, frontier, risk factor, and predictive analyses** to report the prevalence and burden of OA in 2021, the change in trend from 1990 to 2021, the gender difference in the burden of OA, and to project the prevalence of OA and the Age-standardized rate (ASR) of YLDs up to 2035, with the aim of assisting policymakers in all countries and regions to improve OA prevention and control measures.”

**Study Design and Methods**

***(Page 7 Line 13-17)*** **2.1Data Sources and Methods:**..........“The OA data we extracted for GBD 2021 included: **total OA**, **knee osteoarthritis**, **hip osteoarthritis**, **hand osteoarthritis** and **other osteoarthritis**.

This work has been reported under the STrengthening the Reporting of OBservational studies in Epidemiology (STROBE) standard.”

***(Page 8 Line 14-19)*** **2.3 Case Definition:**..........“Consistent with hip and knee osteoarthritis modelling, symptomatic, radiographically confirmed osteoarthritis in any single joint of the hand was used as the reference case definition for hand osteoarthritis, to which alternative case definitions present in the literature were adjusted. Given the paucity of survey data on other osteoarthritis joint sites, US insurance claims data from 2000 to 2016 constituted the sole source of other osteoarthritis data.”

**Results:**

***(Page 19 Line 11-22 & Page 20 Line 1-4)*** **3.4 Risk Factor Analysis for OA**..........“Within the GBD framework, **metabolic risk includes:** high blood glucose, high blood pressure, low bone density, high LDL cholesterol, high BMI, and impaired kidney function. After we included all the above factors in our analysis, according to the output of GBD (https://vizhub.healthdata.org/gbd-results/): high BMI and total metabolic risk were the only two risk factors. These factors were chosen because: **high blood glucose, which may cause accumulation of glycosylation end products leading to inflammation and cartilage damage; high blood pressure, which may be associated with intra-articular vasculopathy, affects cartilage nutrient supply; low bone mineral density, which may alter the biomechanics of the joints and increase the cartilage stress; high LDL cholesterol, which may promote inflammation and oxidative stress; high BMI, which increases the mechanical load on joints with the concomitant release of adipose tissue inflammatory factors; impaired renal function may affect calcium and phosphorus metabolism, leading to abnormal cartilage mineralization**. Dong also showed that the High BMI and Inflammation-Related cohort had the highest risk of osteoarthritis and death, which was associated with obesity-related genetic variants.”

**Discussion:**

***(Page 25 Line 3-17)*** **Discussion**..........“Specifically: 1.Weight Management and Healthy Diet Education: women are advised to maintain a healthy weight to avoid excessive weight gain. 2.Exercise Interventions and Joint Protection: regular physical activity can help women strengthen muscle power, improve joint stability, and slow the progression of osteoarthritis. 3.Hormone Replacement Therapy (HRT): estrogen supplementation helps maintain bone density and joint health. 4. Joint Protection Education: to teach women how to reduce joint strain through proper posture and movement techniques. 5.Nutritional Supplementation and Pharmacological Prevention: adequate supplementation with calcium and vitamin D helps maintain bone strength, reduce the risk of osteoporosis. 6.Mental Health and Stress Management: Maintaining a positive mindset and practicing stress management techniques can help lower immune responses and inflammation levels triggered by mental stress. 7.Early Screening and Prevention: regular medical check-ups to assess joint function, body mass index, and early signs of joint damage allow for timely interventions to prevent OA progression.”

***(Page 29 Line 7-19)*** **Discussion**......“To address the limitations outlined and advance osteoarthritis (OA) burden assessment, future studies should prioritize the following directions:1.Standardization of diagnostic criteria and data collection protocols across low- and middle-income countries to mitigate underdiagnosis and misclassification. 2.Integration of real-time data streams (e.g., electronic health records, wearable sensors) with GBD modeling frameworks to reduce temporal lag and improve responsiveness to emerging risk factors like obesity epidemics. 3.Mechanistic studies on metabolic-inflammatory crosstalk in OA pathogenesis, particularly exploring therapeutic targets. 4.Cost-effectiveness analyses of precision interventions, combining disease-modifying therapies with socioeconomic burden models to guide global resource allocation. These efforts would synergize with the GBD framework to bridge evidence gaps and translate epidemiological insights into actionable clinical strategies.”

Finally, we would like to thank you once again for your critical feedback, which has provided us with new perspectives. The revised version aims to help readers better understand the novel findings of our study. We would be delighted to make additional revisions if you believe there are still areas requiring further elaboration or modification to better distinguish our work from previous studies.

**References**

[1] Bollyky, T. J. et al. Pandemic preparedness and COVID-19: an exploratory analysis of infection and fatality rates, and contextual factors associated with preparedness in 177 countries, from Jan 1, 2020, to Sept 30, 2021. Lancet 399, 1489–1512 (2022).

[2] Friedman, J. et al. Predictive performance of international COVID-19 mortality forecasting models. Nat. Commun. 12, 2609 (2021).

[3] Murray, C. J. & Lopez, A. D. Alternative projections of mortality and disability by cause 1990–2020: Global Burden of Disease Study. Lancet 349, 1498–1504 (1997).

[4] Murray, C.J.L. The Global Burden of Disease Study at 30 years. Nat Med 28, 2019–2026 (2022).

[5] Dong H, Shi Y, Ma Y, et al. Novel metabolic and inflammatory stratification of overweight/obesity to characterize risks of adverse outcomes: A large population-based cohort study. Diabetes Obes Metab. 2025 Feb 19.

At last, on behalf of all the authors, I would like to thank the editors and reviewers once again for your hard work and professional advice, as your meticulous attitude and constructive comments have greatly improved the quality of our research.

Yours sincerely,

All authors

2025-03-26
