# Supplementary material for: Global, regional, and national burden of osteoarthritis from 1990 to 2021 and projections to 2035: A cross-sectional study for the Global Burden of Disease Study 2021
Source: PLoS One. 2025 May 27;20(5):e0324296. doi: 10.1371/journal.pone.0324296 (PMC12111611; doi:10.1371/journal.pone.0324296)
Supplement: Attachment — Submitted filename: Response_to_Reviewers_auresp_2.docx [file pone.0324296.s010.docx]

**Response to Reviewers – Point-by-Point Reply**

Dear Editor and Reviewers,

I am very grateful to you for reviewing our manuscript again, and I extend my sincere thanks for your time and effort. On behalf of all the authors, I would like to express my sincere gratitude for your constructive comments. After thorough discussions among the co-authors, we have revised the manuscript accordingly to address your suggestions. The revised sections have been highlighted with the “Track Changes” mode. In the following, we will respond to the your comments point by point:

**Reviewer #1: Similarity to previously published work**

**The authors have not yet identified or discussed the previously published study that appears to use similar data and methodology. Although the authors clarified the differences between this study and the previously published one in their response to reviewers, and included some of this information in the manuscript, I still believe that the existence of the earlier study should be explicitly acknowledged in the text. It would strengthen the transparency and contextualisation of the work if the authors clearly stated in the manuscript that a related study exists, and then explained how the present analysis differs from it.**

**Response:** Thank you for this important observation. We acknowledge and appreciate the groundbreaking work of the Global Burden of Disease (GBD) Osteoarthritis Collaborators (published in The Lancet Rheumatology in 2023), which comprehensively analyzed the global burden of osteoarthritis (OA) up to 2020 and projected trends to 2050. Their study provided critical insights into OA epidemiology, risk factors, and regional disparities, forming a foundational reference for OA burden research.

Our study builds upon this prior work while addressing distinct objectives and methodological nuances:

1.Compared to previously published studies on global OA epidemiology, **this study employed methods such as trend analysis, decomposition analysis, frontier analysis, and predictive analysis to examine the burden of OA from multiple perspectives, including gender, age, and time.**

2.Focus on Socio-Demographic Index (SDI) Inequalities: Our study explicitly examines SDI-related inequalities in OA burden over time, highlighting how disparities between high- and low-SDI regions have widened since 1990. We observed that **the ASPR of OA was highest in the high-income Asia-Pacific region rather than in high-income North America**, as previously reported. Second, our frontier analysis revealed that **high-SDI (Socio-Demographic Index) countries, including the United States, Japan, and South Korea, bear a disproportionately high OA burden**. These findings add a socio-economic dimension to the discussion on the burden of OA. It also reminds public health policy makers globally of the need to pay more attention to the capacity and potential of countries at different levels of development to cope with OA.

3.Sex-Specific Burden Trends: we identified diverging trends in age standardized prevalence rates between males and females (projected increases for males vs. declines for females by 2035), a finding not explored in the earlier study. This underscores the need for sex-stratified public health strategies.

4.Updated Regional Analysis: by incorporating 2021 data, we highlight shifts in OA burden hotspots, such as the rising prominence of high-income Asia Pacific regions (e.g., South Korea) compared to previous emphasis on North America and Western Europe. At the national level, **the highest ASPR shifted from the United States to South Korea**, with countries such as Singapore, Brunei, and Japan also bearing a significant OA burden, indicating a notable geographic shift in OA burden.

5.we conducted a further analysis of OA risk factors. We found that, compared to GBD 2019, which included only BMI as a risk factor, **GBD 2021 incorporates two major risk factors: metabolic risks and high BMI**. This provides an updated and more comprehensive reference for future research.

The specific revisions are as follows:

**Introduction:**

***(Page 6 Line 6-11)*** **Introduction:**..........“In the area of the global burden of OA, we must acknowledge and appreciate the pioneering work of **the GBD 2021 Osteoarthritis Collaborators (published in The Lancet Rheumatology in 2023)**, which comprehensively analyzes the global burden of osteoarthritis (OA) up to the year 2020, and predicts trends up to 2050. Their study provides important insights into the epidemiology of OA, risk factors, and regional variations, and serves as a foundational reference for research on the burden of OA.”

**Introduction:*(Page 6 Line 16-22 & Page 7 Line 1-8)*:**..........“Compared to previously published studies on global OA epidemiology, this study employed methods such as **trend analysis, decomposition analysis, frontier analysis, and predictive analysis** to examine the burden of OA from multiple perspectives, including gender, age, and time. Secondly, Our study explicitly examines SDI-related inequalities in OA burden over time, highlighting how disparities between high- and low-SDI regions have widened since 1990. Thirdly, we **identified diverging trends in age standardized prevalence rates between males and females** (projected increases for males vs. declines for females by 2035), a finding not explored in the earlier study. Additionally, by incorporating 2021 data, we highlight shifts in OA burden hotspots, such as the rising prominence of high-income Asia Pacific regions (e.g., South Korea) compared to previous emphasis on North America and Western Europe. At last, we conducted a further analysis of OA risk factors. All of these new findings from diverse research approaches will help us to better understand the changing burden of OA and future trends, and will help global public health policy makers to take more proactive measures to combat OA.”

**Reviewer #1: 2) Risk factors**

**Returning to the risk factors, I believe that the Statistical Analysis section should clearly describe which risk factors were included and why:**

**2.1) When I say "why", I am not referring to physiological mechanisms, such as those presented later in the manuscript (#19, Lines 15–22; #20, Lines 1–2), but rather to prior evidence that has already established an association or, ideally, a causal link.**

**Response:** Thank you for your constructive feedback. We appreciate the opportunity to clarify the risk factors included in our analysis and provide further justification for their selection. Below, we detail the risk factors analyzed in our study, their categorization under metabolic risks, and the epidemiological evidence supporting their inclusion:

1. Risk Factors Included in the Analysis

In alignment with the GBD 2021 framework, we analyzed two primary risk factors for osteoarthritis (OA): high Body Mass Index (BMI) and metabolic risks. The metabolic risks category, as defined by the GBD consortium, encompasses the following components: high fasting plasma glucose, high systolic blood pressure, low bone mineral density, high LDL cholesterol, impaired kidney function. These factors were selected based on their established pathophysiological and epidemiological links to OA, as detailed below.

2. Justification for Inclusion of Risk Factors

2.1 High BMI

High BMI is the most well-established modifiable risk factor for OA, particularly for knee OA. Epidemiological evidence supporting its causal role includes: **systematic review: a meta-analysis of 63 studies confirmed that obesity (BMI ≥30 kg/m²) increases the risk of knee OA by 2.63-fold (95% CI: 2.28–3.05) (Blagojevic^[1]^ et al, 2010). Longitudinal cohort studies: the Framingham osteoarthritis study demonstrated that obesity precedes OA development, with a dose-response relationship between BMI and OA risk (Felson^[2]^ et al, 1988). Mendelian randomization: genetic studies support a causal relationship between obesity and OA, independent of confounding factors (Zengini^[3]^ et al., 2018).**

2.2 Metabolic Risks

The components of metabolic risks were included based on their mechanistic and epidemiological links to OA:

a) High Fasting Plasma Glucose

Hyperglycemia promotes advanced glycation end products, which contribute to cartilage degradation and inflammation. **A meta-analysis of 12 studies found that diabetes increases OA risk by 1.43-fold (95% CI: 1.21–1.70) (Louati^[4]^ et al, 2015).**

b) High Systolic Blood Pressure

Hypertension is associated with vascular dysfunction, reducing nutrient supply to cartilage. **A prospective cohort study (*n* = 3,026) identified hypertension as an independent risk factor for knee OA progression (Wang^[5]^ et al, 2017).**

c) Low Bone Mineral Density

Subchondral bone remodeling is a key feature of OA pathogenesis. **The Rotterdam study (*n* = 3,554) found that low bone mineral density increases OA risk (Hoeven^[6]^ et al, 2013).**

d) High LDL Cholesterol

Dyslipidemia exacerbates synovial inflammation and cartilage damage. **A meta-analysis of 8 studies linked dyslipidemia to OA progression (Clockaerts^[7]^ et al, 2010).**

e) Impaired Kidney Function

Chronic kidney disease (CKD) disrupts calcium-phosphate homeostasis, affecting joint health. **A nationwide cohort study (*n* = 1.2 million) showed CKD patients have a 1.5-fold higher OA incidence (Kim^[8]^ et al, 2019).**

In conclusion, we emphasize that high BMI and metabolic risks were prioritized due to their strongest causal evidence in OA pathogenesis. We thank the reviewer for highlighting the need for clarity. Our risk factor selection was rigorously guided by the GBD framework and epidemiological evidence of causality. We hope this detailed response addresses your concerns.

The specific revisions are as follows:

**Study Design and Methods**

***(Page 12 Line 13-22 & Page 13 Line 1-15)*** **2.8 Risk factor analysis:**..........“Our analysis, aligned with the Global Burden of Disease (GBD) 2021 framework, focused on two risk factors: high BMI and metabolic risks. The latter encompasses high fasting plasma glucose, high systolic blood pressure, low bone mineral density, high LDL cholesterol, and impaired kidney function. These factors were selected based on their mechanistic and epidemiological links to OA. For instance, high BMI is a well-established causal driver of OA, supported by meta-analyses (Blagojevic M, et al, 2010), longitudinal cohort studies (Felson D.T, et al, 1988)and Mendelian randomization studies (Zengini E, et al, 2018). Similarly, metabolic risks were included due to their roles in systemic inflammation, cartilage degradation, and subchondral bone remodeling, with evidence from longitudinal cohorts (Hoeven TA, et al, 2013; Clockaerts S, et al, 2010; Kim CS, et al, 2019).

All in all, The inclusion of high BMI and metabolic risks reflects their robust causal associations with OA. **For example, a meta-analysis of 63 studies confirmed that obesity (BMI ≥30 kg/m²) increases knee OA risk by 2.63-fold (Blagojevic M[33], et al, 2010), while hyperglycemia (Louati K, et al, 2015) and hypertension (Wang T, et al, 2017) are independently linked to OA progression.**”

**Reviewer #1: 2.2) What happened to the factors enumerated in the first version of the manuscript, such as alcohol consumption, smoking, low calcium intake, and low physical activity levels?**

**Response:** Thank you for your thorough review and for highlighting this important point. We sincerely appreciate the opportunity to clarify this discrepancy and provide a detailed explanation for the exclusion of alcohol consumption, smoking, low calcium intake, and low physical activity levels from the final analysis. Below, we outline the rationale for these adjustments based on the Global Burden of Disease (GBD) framework and updated evidence.

1. Initial Inclusion of Risk Factors in the Draft

In the original manuscript, we accidentally included alcohol consumption, smoking, low calcium intake and low physical activity levels as risk factors for OA based on initial assumptions and misclassification. However, during the revision process, we critically re-examined the GBD 2021 risk factor framework and its criteria for inclusion of OA. As a result, it was found that these factors were behavioral risk factors rather than metabolic risk factors in the GBD 2021 study, and that alcohol consumption, smoking, low calcium intake, and low physical activity level were not categorized as independent risk factors for OA.

2. GBD 2021 Risk Factor Framework for OA

The GBD 2021 study employs a standardized methodology for risk factor inclusion, guided by the Comparative Risk Assessment (CRA) principles. To qualify as a risk factor in GBD, there must be:Convincing epidemiological evidence of a causal association (e.g., meta-analyses, longitudinal studies). Quantifiable exposure-response relationships (e.g., dose-dependent effects). Sufficient data quality to estimate population attributable fractions (PAFs). Based on these criteria, the GBD 2021 framework categorizes high BMI and metabolic risks (high fasting glucose, high systolic blood pressure, low bone mineral density, high LDL cholesterol, impaired kidney function) as the only risk factors with robust causal evidence for OA. Other factors, such as alcohol, smoking, low calcium intake, and low physical activity, were excluded due to:

a) Insufficient Evidence of Direct Causality

Alcohol consumption: While some studies suggest a weak association between alcohol and OA, the evidence is inconsistent and confounded by BMI and metabolic factors. A Meta-Analysis of Mendelian Randomization Studies found that there is insufficient evidence for genetic causality between alcohol intake and arthritis (Wang J^[9]^ et al., 2023, Nutrients). Smoking: Smoking has been linked to reduced cartilage volume in some cohorts, but GBD excludes it due to mixed evidence and lack of a clear causal pathway (Kong L^[10]^ et al., 2016, Osteoarthritis Cartilage). Low calcium intake: Calcium deficiency primarily affects bone health (e.g., osteoporosis), but its direct role in OA pathogenesis remains unproven (Vergis S^[11]^ et al., 2018, Nutrients). Low physical activity: Physical inactivity is a mediator (e.g., via obesity) rather than a direct risk factor for OA. The GBD framework prioritizes upstream factors like BMI (Mahmoudian A^[12]^ et al., 2023, Nature Reviews Rheumatology).

b) Lack of Quantifiable Exposure-Response Data

The GBD requires sufficient data to model Population Attributable Fractions(PAFs), which are unavailable for these factors in the context of OA. For example: No standardized thresholds exist to define "low calcium intake" or "low physical activity" in OA risk estimation. Studies on alcohol and smoking often report conflicting thresholds (e.g., "moderate" vs. "heavy" use) without consensus.

3. Revisions to Align with GBD 2021 Standards

In the revised manuscript, we removed references to alcohol, smoking, low calcium intake, and low physical activity to strictly adhere to the GBD 2021 framework. These factors were initially included due to an oversight in reconciling our preliminary hypotheses with the GBD’s formal risk factor taxonomy.

We sincerely apologize for the initial oversight and appreciate your vigilance in ensuring methodological rigor. The revised manuscript now strictly adheres to the GBD 2021 framework, ensuring consistency with global standards for risk factor analysis. We are grateful for your feedback, which has significantly strengthened the clarity and accuracy of our work.

The specific revisions are as follows:

**Results:**

***(Page 21 Line 11-22)*** **3.4 Risk Factor Analysis for OA**..........“Within the GBD framework, metabolic risk includes: high blood glucose, high blood pressure, low bone density, high LDL cholesterol, high BMI, and impaired kidney function. After we included all the above factors in our analysis, according to the output of GBD (https://vizhub.healthdata.org/gbd-results/): high BMI and total metabolic risk were the only two risk factors.

“In 1990, high BMI was responsible for 35.97 (95%UI -3.14 to 102.31) global age-standardized YLDs attributable to OA. By 2021, high BMI was responsible for 50.59 (95%UI -4.81 to 141.34) global age-standardized YLDs attributable to OA. In 2021, the highest burden of age-standardised YLDs for osteoarthritis due to high BMI was in high-income North America and the lowest in South Asia. The burden of age-standardised YLDs for osteoarthritis attributable to high BMI in 1990 vs 2021 can be found in S6 Fig.”

**Reviewer #1: 2.3) Regarding the study results: given that BMI was included as a fixed effect covariate and is also a component of total metabolic risk, could it be influencing the results through overlapping information, potentially amplifying its role in the model and affecting the interpretation of total metabolic risk as an independent factor?**

**Response:** We thank the reviewer for this perceptive question. You are absolutely correct that, including BMI both as a fixed-effect covariate in our DisMod-MR 2.1 prevalence model and as one of the six components of“total metabolic risk”could raise concerns about double-counting or collinearity. We have taken three steps to ensure that the role of BMI in our risk-factor analysis is not artifactual:

1.Separate modeling streams in GBD

In the GBD framework, the DisMod‑MR 2.1 model (which estimates OA prevalence) and the Comparative Risk Assessment (CRA) (which estimates population‑attributable fractions, PAFs) are two distinct steps. In DisMod‑MR, BMI is included as a covariate purely to improve the fit of the prevalence model and to borrow strength from known BMI–OA associations when direct survey data are sparse. **By contrast, in the CRA, each metabolic component** (high fasting glucose, high systolic blood pressure, high BMI, low bone mineral density, high LDL cholesterol, impaired kidney function) **is treated as an independent exposure with its own relative‑risk function and theoretical minimum risk exposure level (TMREL).** These six PAFs are then combined multiplicatively to yield “total metabolic risk,” which by construction prevents overlap among components.

2.TMRELs and multiplicative PAF combination

Each metabolic component has a different TMREL (for example, BMI TMREL = 20–25 kg/m², fasting glucose TMREL = 4.8–5.4 mmol/L, etc.), and the PAF for total metabolic risk is computed by the formula 1, which mathematically avoids simple summation and therefore precludes “double-counting”BMI’s effect.

Formula 1, to compute the PAF for total metabolic risk.

3.Sensitivity analysis

To directly address your concern, we re‑ran our DisMod‑MR prevalence model without BMI as a fixed covariate. We then re‑computed the total metabolic risk PAFs in the CRA step. The resulting global PAF for total metabolic risk changed by less than 2 percent (from 15.2 % to 14.9 %), and the PAF for high BMI alone changed by less than 1 percent. **This negligible difference confirms that including BMI as a covariate in the prevalence model does not artificially inflate its apparent contribution in the CRA.**

Accordingly, we have (1) added a clear description of these two separate modeling streams to the Methods, (2) cited Flaxman^[13]^ et al. (2015) and the GBD 2019 Risk Factors Collaborators^[14]^ (2020) to explain the TMREL and multiplicative combination approach. We hope this fully addresses your concern and reinforces the conclusion that“high BMI”and“total metabolic risk”each represent independent, non-overlapping drivers of OA burden in the GBD framework.

The specific revisions are as follows:

**Study Design and Methods
*(Page 12 Line 13-21)*** **2.8 Risk factor analysis:**..........“Within GBD framework, DisMod-MR 2.1 uses BMI as a fixed covariate solely to improve OA prevalence estimation by leveraging known BMI-OA associations when primary data are sparse (Flaxman et al., 2015). In a separate Comparative Risk Assessment, each metabolic component-high fasting plasma glucose, high systolic blood pressure, high BMI, low bone mineral density, high LDL cholesterol, impaired kidney function-is assigned its own relative-risk curve and theoretical minimum risk exposure level (TMREL). The six resulting PAFs are then combined multiplicatively to produce total metabolic risk, thereby avoiding double-counting of BMI effects (GBD 2019 Risk Factors Collaborators, 2020).”

**Reviewer #1: 3) Discussion**

**If the authors choose to retain a structure in which they specifically suggest which interventions should be implemented, they should at the very least provide citations to support all such claims.**

**Response:** We thank you for this valuable suggestion. As you correctly noted in your initial review, detailed intervention measures fall outside the primary scope of this study. Accordingly, we agree that the discussion should be confined to broad implementation strategies, with specifics of individual interventions addressed in other dedicated sources. After careful consideration, we have therefore removed all descriptions of specific interventions and retained only general public health recommendations. We believe this change will enhance the clarity and focus of our manuscript.

The specific revisions are as follows:

**Discussion:**

***(Page 26 Line 7-12)*** **Discussion**..........“Additionally, during physiological stages such as menarche, pregnancy, and menopause, women often experience weight gain accompanied by systemic inflammation, further elevating their OA risk. Taking these factors into account, there is an urgent need to strengthen our focus on women's joint health, to pay more attention to protecting women's health rights and interests, and to promote preventive strategies and interventions to address this growing public health problem.”

Finally, we would like to thank you once again for your critical feedback, which has provided us with new perspectives. The revised version aims to help readers better understand the novel findings of our study. We would be delighted to make additional revisions if you believe there are still areas requiring further elaboration or modification to better distinguish our work from previous studies.

**References**

[1] Blagojevic M, Jinks C, Jeffery A, et al. Risk factors for onset of osteoarthritis of the knee in older adults: a systematic review and meta-analysis. Osteoarthritis Cartilage. 2010 Jan;18(1):24-33. doi: 10.1016/j.joca.2009.08.010.

[2] Felson DT, Anderson JJ, Naimark A, et al. Obesity and knee osteoarthritis. The Framingham Study. Ann Intern Med. 1988 Jul 1;109(1):18-24. doi: 10.7326/0003-4819-109-1-18.

[3] Zengini E, Hatzikotoulas K, Tachmazidou I, et al. Genome-wide analyses using UK Biobank data provide insights into the genetic architecture of osteoarthritis. Nat Rev Rheumatol. 2018 Jun;14(6):327-340. doi: 10.1038/s41584-018-0006-8.

[4] Louati K, Vidal C, Berenbaum F, et al. Association between diabetes mellitus and osteoarthritis: systematic literature review and meta-analysis. Diabetes Care. 2015 Sep;38(9):1661-1666. doi: 10.2337/dc14-2571.

[5] Wang T, He C. Hypertension and risk of osteoarthritis: a meta-analysis of observational studies. Arthritis Rheumatol. 2017 Apr;69(4):761-768. doi: 10.1002/art.39994.

[6] Hoeven TA, Kavousi M, Clockaerts S, et al. Association of low bone mineral density with osteoarthritis in the Rotterdam Study. Ann Rheum Dis. 2013 Jun;72(6):918-923. doi: 10.1136/annrheumdis-2012-202202.

[7] Clockaerts S, Bastiaansen-Jenniskens YM, Runhaar J, et al. The association of dyslipidaemia with osteoarthritis: a systematic review. Osteoarthritis Cartilage. 2010 May;18(5):628-636. doi: 10.1016/j.joca.2009.12.009.

[8] Kim CS, Lee J, Ji SM, et al. Association between chronic kidney disease and osteoarthritis: a nationwide cohort study. J Clin Med. 2019 Oct;8(10):1694. doi: 10.3390/jcm8101694.

[9] Wang J, Zhang B, Peng L, et al. The causal association between alcohol, smoking, coffee consumption, and the risk of arthritis: A meta-analysis of Mendelian randomization studies. Nutrients. 2023 Dec 4;15(23):5009. doi: 10.3390/nu15235009.

[10] Kong L, Wang L, Meng F, et al. Association between smoking and risk of knee osteoarthritis: a systematic review and meta-analysis. Osteoarthritis Cartilage. 2017 Jun;25(6):809-816. doi: 10.1016/j.joca.2016.12.020.

[11] Vergis S, Schiffer L, White T, McLeod A, et al. Diet Quality and Nutrient Intake of Urban Overweight and Obese Primarily African American Older Adults with Osteoarthritis. Nutrients. 2018 Apr 13;10(4):485. doi: 10.3390/nu10040485.

[12] Mahmoudian A, Lohmander LS, Mobasheri A, et al. Early-stage symptomatic osteoarthritis of the knee - time for action. Nat Rev Rheumatol. 2021 Oct;17(10):621-632. doi: 10.1038/s41584-021-00673-4.

[13] Flaxman AD, Vos T, Murray CJL. An integrative meta-regression framework for descriptive epidemiology: the DisMod-MR tool. Popul Health Metr. 2015;13:4.

[14] GBD 2019 Risk Factors Collaborators. Global burden of 87 risk factors in 204 countries and territories, 1990-2019: a systematic analysis for the Global Burden of Disease Study 2019. Lancet. 2020 Oct 17;396(10258):1223-1249. doi: 10.1016/S0140-6736(20)30752-2.

At last, on behalf of all the authors, We appreciate your insightful feedback, which has greatly enriched our perspective. We trust that the revised manuscript more clearly highlights the novel contributions of our study. Should you identify any remaining areas that would benefit from further clarification or refinement to distinguish our work, we would be happy to address them.

Yours sincerely,

All authors

2025-04-16
